# Immunological effect of irreversible electroporation on hepatocellular carcinoma

**DOI:** 10.1186/s12885-021-08176-x

**Published:** 2021-04-21

**Authors:** Xiaoxia Guo, Fang Du, Qin Liu, Yan Guo, Qingbing Wang, Wei Huang, Zhongmin Wang, Xiaoyi Ding, Zhiyuan Wu

**Affiliations:** 1 Department of Interventional Radiology, Ruijin Hospital, Shanghai Jiao Tong University School of Medicine, Shanghai, 200025 China; 2 Department of Rheumatology, Renji Hospital, Shanghai Jiao Tong University School of Medicine, Shanghai, 200025 China; 3 Department of Pathology, Ruijin Hospital, Shanghai Jiao Tong University School of Medicine, Shanghai, 200025 China

**Keywords:** Irreversible electroporation, Hepatocellular carcinoma, Tumor ablation, Immunological effect, Antitumor immune response

## Abstract

**Background:**

This study intends to investigate the immunological effects of tumor ablation with irreversible electroporation (IRE).

**Methods:**

We evaluated the systemic immune response in patients with hepatocellular carcinoma (HCC) after IRE treatment. Furthermore, we analyzed the tumor infiltrating T lymphocytes and the level of serum cytokines in IRE and control groups of tumor-bearing mice.

**Results:**

We observed that IRE induced an increase in WBC, neutrophil and monocyte counts and a decrease in lymphocyte count 1 day post-IRE and returned to baseline values within 7 days in the patients. Meanwhile, circulating CD4^+^ T cell subsets, but not CD8^+^, decreased 1 day post-IRE. The activated T cells and natural killer (NK) cells increased, and regulatory T (Treg) cells decreased. Furthermore, a significant increase in cytotoxic CD8^+^ T cells infiltration was observed on ablative tumors in mice. The level of serum IFN-γ also significantly increased in the IRE group.

**Conclusions:**

Our study demonstrated that IRE upregulated activated T cells and downregulated Tregs in the peripheral blood of patients. Meanwhile, the results from the animal model indicated that IRE could induce antitumor adaptive immunity dominated by the infiltration of cytotoxic CD8^+^ T cells into the tumors, accompanied by reduced Tregs.

## Background

Image-guided tumor ablation therapies such as radiofrequency ablation, microwave ablation and cryoablation are now in widespread clinical use to treat a broad range of benign or malignant solid tumors [1, 2]. Most of these therapies rely on thermal energy to destroy the tumor tissues by inducing coagulation necrosis. Irreversible electroporation (IRE) is a new non-thermal tumor ablation technique that involves the application of high voltage electrical pulses to generate an electric filed across the target tissue. Although the mechanism is not yet completely understood, the electric filed is thought to change the electrochemical potential across the cell membrane, thereby causing instability of the lipid bilayer, which can cause the formation of aqueous pathways or permanent nanopores through the membrane, and the cell loses its homeostatic resulting in cell death [3]. IRE selectively destroys lipid bilayers of the cell membrane, sparing the connective tissue and collagen, therefore, vital structures such as blood vessels or bile ducts can remain intact [4]. Additionally, its efficacy is not impaired by heat sink effects in the treatment of tumors located close to large blood vessels [5, 6]. These advantages make IRE suitable to target tumors that cannot be treated by thermal ablations.

The accumulating evidence from the literature suggest that the host immune response is involved in cancer development and progression; activating antitumor immune response plays a crucial role in cancer control and therapy [7–9]. Since increased permeability of the cell membrane is thought to be the primary mechanism of cell death caused by IRE [10], the substantial native tumor antigens may be exposed, allowing for them to act as in situ vaccines to generate antitumor immune reaction [11]. Meanwhile, intact and persistent microvessels within the IRE ablation zone may facilitate the infiltration of immune cells [12]. While a few studies had reported that IRE did not induce any change in immune cell infiltration, other contradictory reports suggest that IRE provides beneficial immunological effects [13–17]. Although the literature on immune cell recruitment after IRE treatment remain conflicting, there is strong evidence of local and systemic antitumor immune response resulting from IRE.

Based on the contradictory results in these preclinical animal studies, we aimed to further investigate the immunological response to tumor ablation with IRE in patients with solid tumors and mouse models bearing tumors.

## Methods

### Patients

The Ethics Committee of Ruijin Hospital Affiliated to Shanghai Jiao Tong University School of Medicine approved this retrospective study (reference number: AF-0406). Written informed consent was obtained from each patient. From October 2016 to August 2019, 61 patients in our center underwent IRE treatment (including 26 liver tumors, 17 pancreatic tumors, 4 renal tumors, 5 adrenal gland tumors, 6 retroperitoneal tumors; one patient with both liver and pancreatic tumors, and two patients with both liver and retroperitoneal tumors). Due to the immunity of these types of patients were relative to the types of malignancies that were treated were highly variable, we selected a single tumor type--- hepatocellular carcinoma (HCC) for immunological analysis, 3 cholangiocarcinoma and 12 hepatic metastases were excluded. Data were collected on a total of 11 consecutive patients with HCC (8 men, 3 women, mean age, 60.8 ± 9.3 years) in the study. All patients had a history of chronic HBV infection and cirrhosis. No chemotherapy or interferon therapy was received previously. After the diagnosis of HCC, all patients were treated with oral nucleoside antiviral drugs. During the follow up, all patients received no adjunctive therapies.

### IRE procedure in patients and sample collection

Two experienced interventional radiologists performed all the procedures. All patients were administered with muscle relaxants and general anesthesia. IRE was performed using a NanoKnife system (AngioDynamics, Latham, NY, USA) with an electrocardiogram (ECG) synchronization device under the guidance of CT. Nineteen gauge monopolar needles were placed in parallel around the tumors percutaneously at the intervals of 1.2–2.2 cm. Tip exposure of the needles was 1.0–2.0 cm. The number of needles was decided according to the tumor size. The parameters of IRE ablation were set as follows: average electric field intensity, 1500 V/cm; pulse length, 70–90 μs; 90 pulses. The ablation range covered the whole tumor with an ablation margin of at least 5 mm. Peripheral blood samples were collected 1 day before IRE therapy and used as baseline values. Additional blood samples were collected 1 day, 3 days, 7 days, 2 weeks, and 4 weeks after IRE treatment. Blood tests included blood cells analysis and immune cells analysis. A routine clinical flow cytometry test protocol was followed for analyzing immune cells in the peripheral blood. During the IRE treatment and the follow up period, no collateral events related to IRE were observed.

### Cell culture and animal models

All animal studies were performed in accordance with the Guidelines for the Care and Use of Laboratory Animals with approval by the Institutional Animal Care and Use Committee of Shanghai Jiao Tong University School of Medicine prior to initiation of experiments. The mouse hepatic carcinoma cell lines, H22 were purchased from China Center for Type Culture Collection (Wuhan, China). H22 cells were cultured in RPMI containing 10% FBS and 1% penicillin-streptomycin, in an incubator with a humidified atmosphere of 5% CO_2_ at a temperature of 37 °C. H22 cells (5 × 10^6^) were suspended in 200 μL phosphate buffered saline and injected subcutaneously into the right flank of 5- to 6-week-old male BALB/c mice (commercially obtained from LINGCHANG BIOTECH, Shanghai, China). Two weeks after the injection, the diameter of the tumors reached nearly 1 cm.

### IRE procedure in mice and sample collection

After 2 weeks of modeling, a total of 30 mice were randomly divided into two groups: the control group (*n* = 15) and the IRE group (*n* = 15). For the IRE group, the mice were anesthetized by injecting sodium pentobarbital (10 mg/mL, 50 mg/kg body weight) intraperitoneally. Then, each mouse was fixed on an insulating plate, and the IRE procedure was performed using an ECM 830 Square Wave Electroporation system (BTX Harvard Apparatus, Holliston, MA, USA) with a pair of genetrodes (BTX item #45–0161, BTX Harvard Apparatus, Holliston, MA, USA). The genetrodes with a 10 mm gap were inserted into the tumors to deliver electric pulses with the following parameters: voltage, 1200 V; pulse length, 90 μs; 90 pulses. This protocol was selected to produce a complete ablation for the tumors. The mice in the control group received sham procedures with the genetrodes inserted into the tumors but no electric pulses were given. The blood samples and tumor samples were collected at 3, 7, and 14 days post-IRE procedure from five mice separately. Samples from the control group were collected at the same time. At the end of the experiments, the mice were killed by standard CO_2_ asphyxiation.

### Flow cytometry analysis

Tumors isolated from the mice were digested mechanically to obtain single-cell suspensions. The cell suspensions were surface stained with PE-Cy7-labeled anti-CD3 (eBioscience, San Diego, CA, USA), FITC-labeled anti-CD4 (eBioscience, San Diego, CA, USA) and PE-Cy5-labeled anti-CD8 (eBioscience, San Diego, CA, USA) monoclonal antibodies, and then treated with Fixation/Permeabilization Kit (BD Biosciences, Franklin Lakes, NJ, USA). Then, the cells were stained intracellularly with PE-labeled anti-FoxP3 (eBioscience, San Diego, CA, USA) and Pacific Blue-labeled anti-Granzyme B monoclonal antibodies (Biolegend, San Diego, CA, USA). The stained cells were analyzed with CytoFLEX LX flow cytometer (Beckman Coulter, Brea, CA, USA). Data were analyzed using CytExpert software (Beckman Coulter, Brea, CA, USA).

### Lactate dehydrogenase (LDH) cytotoxicity assay

Single-cell suspensions from mice tumors were prepared. T cells were isolated from the single-cell suspensions by negative selection using the Pan T Cell Isolation Kit II (Miltenyi Biotec, Bergisch Gladbach, Germany). The isolated T cells were stimulated with recombinant murine IL-2 (PeproTech, Rocky Hill, NJ, USA) for 3 days. Then, the T cells were added to H22 cells with an effector to target cell ratio of 20:1, and the cells were co-cultured in 96-well plates for 24 h. Evaluation of T cell cytotoxicity activity was performed using an LDH-Cytox™ Assay kit (BioLegend, San Diego, CA, USA), according to the manufacturer’s protocol. The cytotoxicity percentage was calculated as follows: (LDH experimental − LDH spontaneous) / (LDH maximum − LDH spontaneous) × 100%. LDH experimental represents the LDH release activity from the T cells and tumor cells co-culture. Spontaneous LDH release activity was obtained from tumor cells cultured separately. The maximal LDH release activity was obtained following lysis of the tumor cells.

### Immunohistochemistry analysis

The tumor tissue removed from each mouse was fixed in 4% paraformaldehyde and embedded in paraffin for 5 μm-thick sections. After being deparaffinized and rehydrated, the sections were treated with sodium citrate buffer (pH = 6), and the microwave was used for antigen retrieval. The activity of endogenous peroxidase was blocked with 3% H_2_O_2_ in methanol. The sections were then incubated with anti-CD3 monoclonal antibody (Abcam, Cambridge, UK), anti-CD4 monoclonal antibody (Abcam, Cambridge, UK), and anti-CD8 monoclonal antibody (Abcam, Cambridge, UK) at 4 °C overnight, respectively. Afterwards, the sections were stained with HPR-conjugated secondary antibody, and the positive reactions were visualized with diaminobenzidine (DAB). Finally, the sections were counterstained with Mayer’s hematoxylin. Digital images of the stained sections were obtained in five randomly selected fields both at the interior regions and the margin of tumors using a fluorescence microscope. The positive cell numbers were counted and the results from the five areas were averaged and used in the statistical analysis.

### Cytometric bead array (CBA) analysis

Blood serum was separated from the blood sample obtained from the tumor-bearing mouse by centrifugation at 3000 g for 20 min and then stored at − 80 °C, until later analysis. Serum cytokine analysis was performed using the CBA Flex Set (BD Biosciences, Franklin Lakes, NJ, USA), containing mouse IFN-γ, IL-1β, IL-2, IL-10, and TNF-α. Mouse Soluble Protein Flex Set Standards and samples were prepared according to the manufacturer’s instruction. The samples were acquired on the flow cytometer (BD LSRFortessaTM X-20, Franklin Lakes, NJ, USA). The data were analyzed using FCAP Array software (BD Biosciences, Franklin Lakes, NJ, USA).

### RNA sequencing analysis

Tumors from IRE and control groups 7 days postoperative were separated for RNA sequencing analysis. Total RNA was extracted using Trizol reagent (Roche, Basel, Switzerland). Total RNA quality was evaluated on an Agilent 2100 Bioanalyzer (Agilent, Santa Clara, CA, USA). Library preparation was performed from the pooled RNA using an Illumina TruSeq RNA Sample Preparation Kit v2 (Illumina, San Diego, CA, USA) and sequenced on the Illumina HiSeq 4000 platform (Illumina, San Diego, CA, USA). The sequenced reads were aligned to the mouse genome mm10 by HISAT2 [18]. FeatureCounts was used to quantitate the transcriptome, using the GTF annotation files [19]. Differential analyses were performed to the count files using DESeq2 packages, following standard normalization procedures [20]. The differentially expressed genes (DEGs) were identified with *p* values < 0.05 and absolute log2 fold change > 1. Gene Ontology (GO) enrichment analysis was performed using Metascape (http://metascape.org).

### Statistical analysis

Statistical analysis was performed using SPSS statistical software, version 23 (IBM, Armonk, NY, USA). The immunohistochemistry results were analyzed with the Mann-Whitney test. For other analysis, Student’s t-test was used. All data were expressed as mean ± SEM (standard error of the mean) of n independent measurements. GraphPad Prism 7 software (GraphPad, San Diego, CA, USA) was used to plot graphs. *P* < 0.05 was considered statistically significant.

## Results

### An immediate innate immune response was observed after IRE

**Fig. 1:**
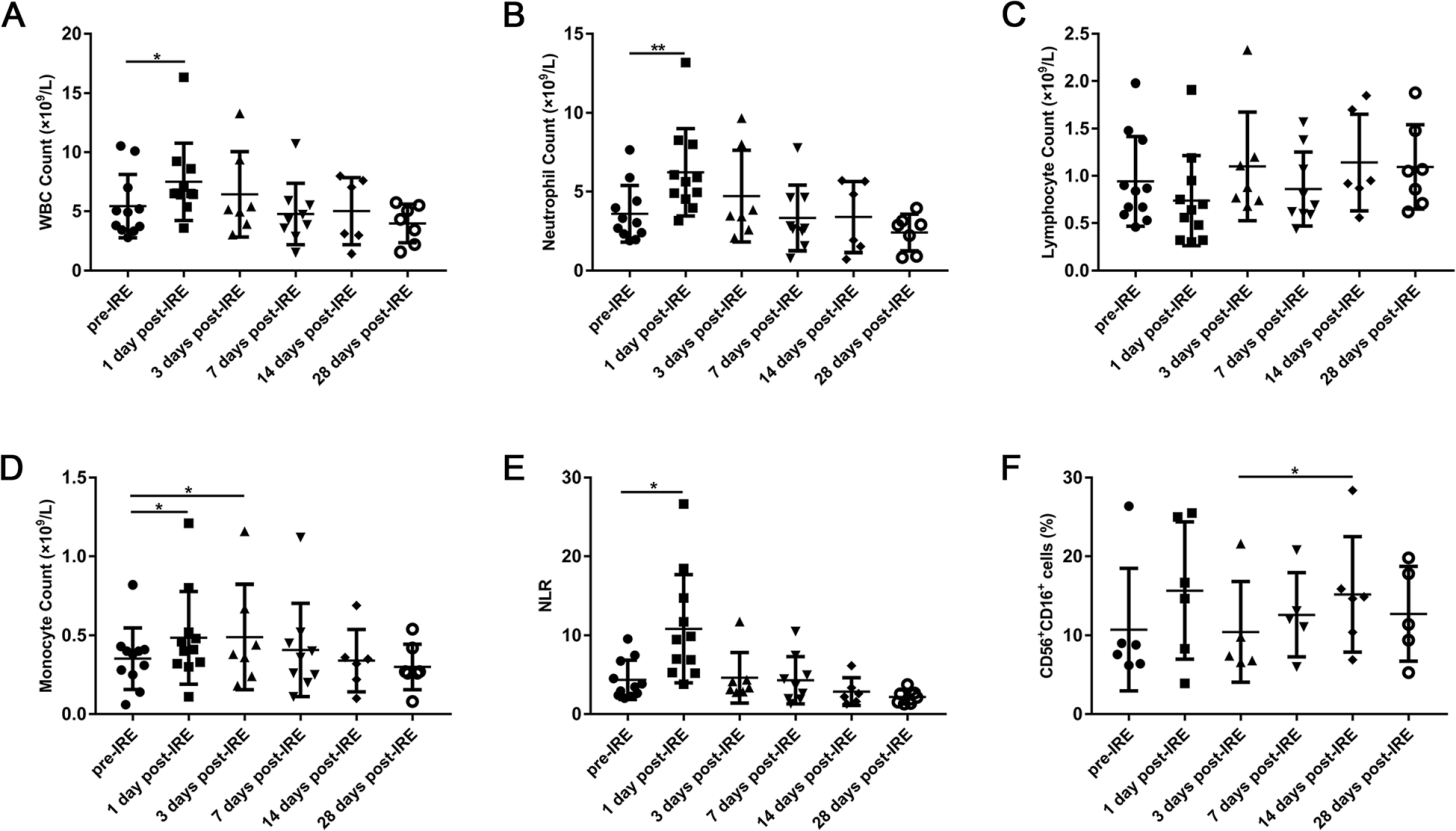
Immune cells in the peripheral blood of patients before IRE treatment (pre), and 1, 3, 7, 14, and 28 days post-IRE. **a-d** WBC, neutrophil, lymphocyte and monocyte counts, **e** neutrophil to lymphocyte ratio (NLR), **f** The percentage of natural killer cells (CD56^+^CD16^+^). ^*^p < 0.05, ^**^p < 0.01

Peripheral blood samples obtained from 11 patients with HCC at six different time points, pre- and post-IRE ablation, were tested for systemic immune reaction. However, complete follow-up information was not obtained from some patients. We applied scatter plots to show the detailed information collected. As shown in Fig. 1, WBC, neutrophil and monocyte counts, as well as neutrophil to lymphocyte ratio (NLR) were found to be elevated significantly 1 day post-IRE; however, they returned to baseline values gradually within 7 days after IRE. On the contrary, lymphocyte count declined 1 day post-IRE, which then increased gradually. Meanwhile, IRE led to a slight and non-significant increase on the percentage of natural killer (NK) cells (CD56^+^CD16^+^) on day 1 (11.4 ± 8.5 vs 15.9 ± 9.7%), followed by a decrease on day 3, then a significant increase was observed from day 3 to day 14 (10.4 ± 6.4 vs 15.3 ± 8.2%, *p* < 0.05) (Fig. 1f).

### IRE led to increased activated T cells and decreased Treg cells

**Fig. 2:**
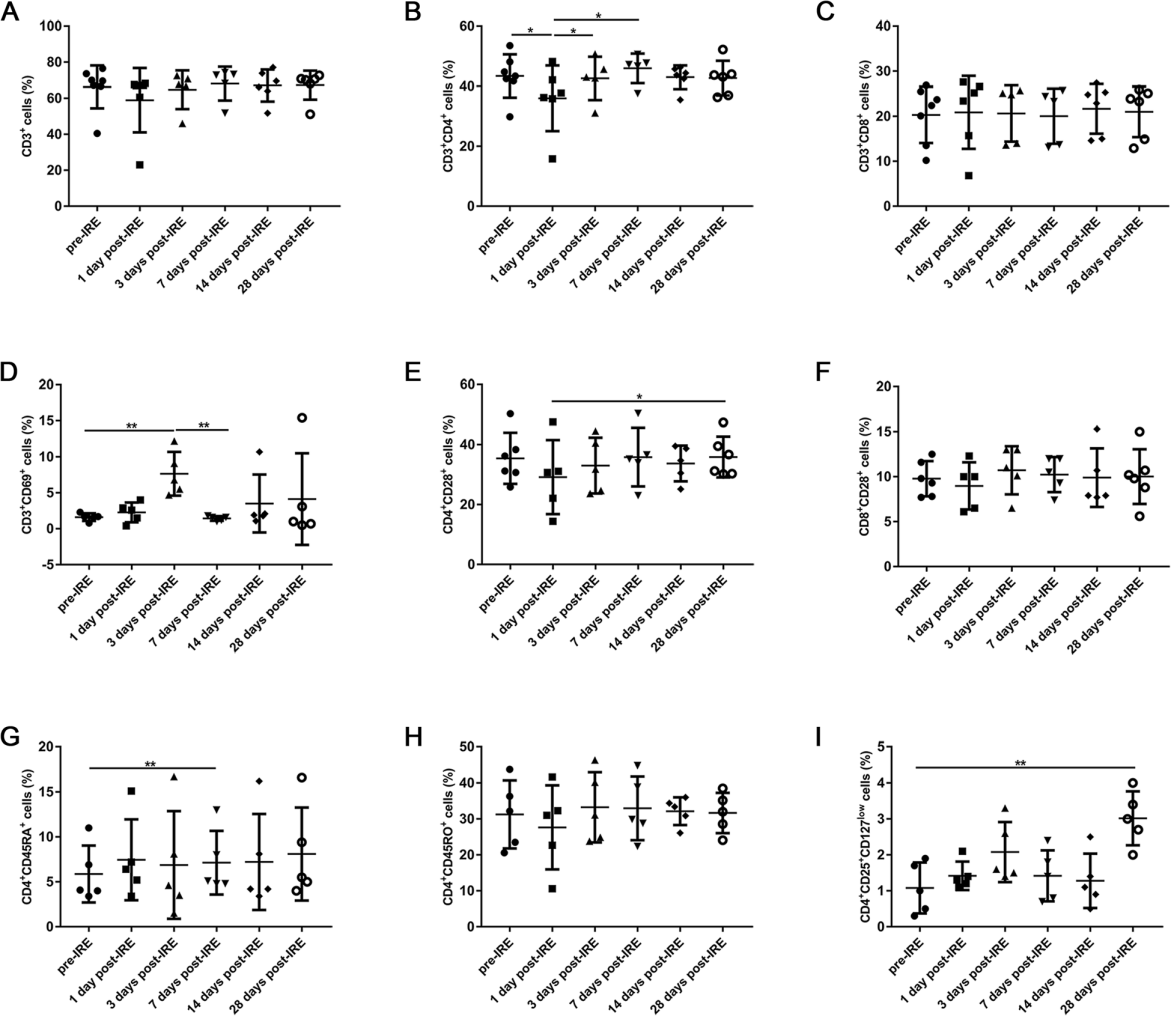
The percentages of immune cells in the peripheral blood of patients before IRE treatment (pre) and 1, 3, 7, 14, and 28 days post-IRE, as analyzed with flow cytometry: **a** CD3^+^ T cells, **b** CD3^+^CD4^+^ T cells, **c** CD3^+^CD8^+^ T cells, **d** Activated T cells (CD3^+^CD69^+^), **e** Activated CD4^+^ T cells (CD4^+^CD28^+^), **f** Activated CD8^+^ T cells (CD8^+^CD28^+^), **g** CD4^+^ naïve cells (CD4^+^CD45RA^+^), **h** CD4^+^ memory cells (CD4^+^CD45RO^+^), and **i** Regulatory T cells (CD4^+^CD25^+^CD127^low^). ^*^p < 0.05, ^**^p < 0.01

The lymphocytes profile data from all the patients were analyzed, the results showed that compared to baseline levels, the percentages of CD3^+^ (Fig. 2a) and CD3^+^CD4^+^ (Fig. 2b) T cells were decreased immediately after IRE (day 1), followed by a steady increase in the next days, as did the activated (CD4^+^ CD28^+^) (Fig. 2e) and memory (CD4^+^CD45RO^+^) (Fig. 2h) CD4^+^ T cells. Meanwhile, the patients showed a steady increase in the percentage of CD4^+^ naïve cells (CD4^+^CD45RA^+^) (Fig. 2g). However, the percentages of CD3^+^CD8^+^ T cells (Fig. 2c), as well as its activated subset (CD8^+^ CD28^+^) (Fig. 2f) remained unchanged. Moreover, the percentage of activated T cells (CD3^+^CD69^+^) increased significantly 3 days post-IRE (1.6 ± 0.5 vs 7.7 ± 3.0%, *p* < 0.01), followed by a decrease on day 7 (Fig. 2d). The trend for regulatory T (Treg) cells (CD4^+^CD25^+^CD127^low^) was similar to activated T cells at the beginning, a decrease was observed from day 3 to day 14 (2.1 ± 0.8 vs 1.3 ± 0.8%), but a significant increase was induced 1 month after IRE (1.1 ± 0.7 vs 3.0 ± 0.8%, *p* < 0.01) (Fig. 2i).

### Cytotoxic CD8^+^ T cells increased but Treg decreased in mice tumor after IRE

**Fig. 3:**
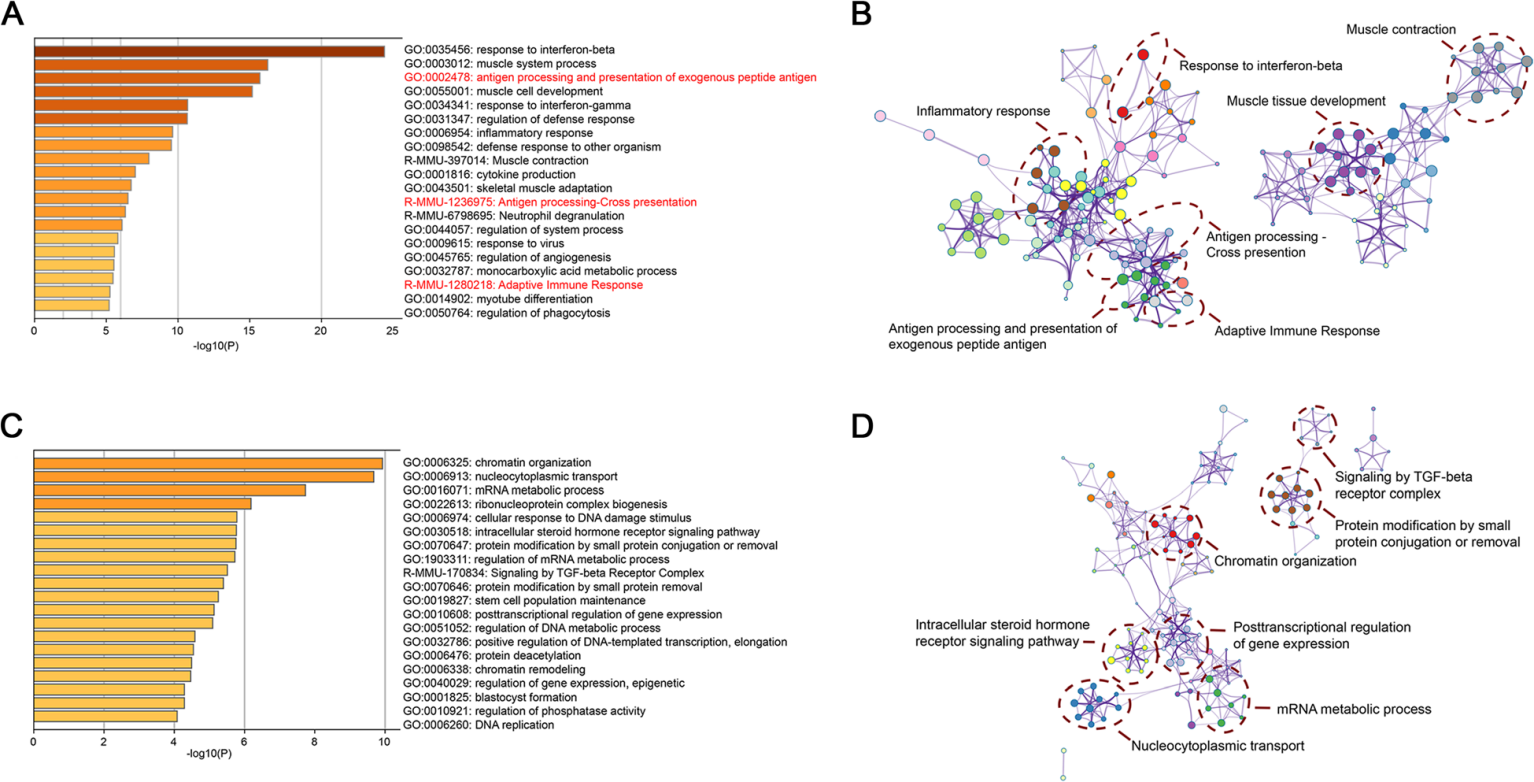
Gene expression profiling in the tumors of mice in the IRE and control groups 7 days post-IRE obtained from RNA sequencing. **a** Heatmap displaying enrichment of DEGs up-regulated. Adaptive immune process related pathways are highlighted in red. **b** Enriched ontology clusters of DEGs up-regulated. **c** Heatmap displaying enrichment of DEGs down-regulated. **d** Enriched ontology clusters of DEGs down-regulated

To probe whether the IRE treatment resulted in significant up-regulation of pathways associated with the adaptive immune process, RNA-seq expression was performed in IRE treated mice and controls. The results of GO analysis indicated that several adaptive immune process related pathways were up-regulated in the IRE treatment group, including antigen processing and presentation of exogenous peptide antigen, antigen processing-cross presentation, and adaptive immune response (Fig. 3a, b). Additionally, several pathways, such as signaling by TGF-beta Receptor Complex were down-regulated in IRE treatment group (Fig. 3c, d).

**Fig. 4:**
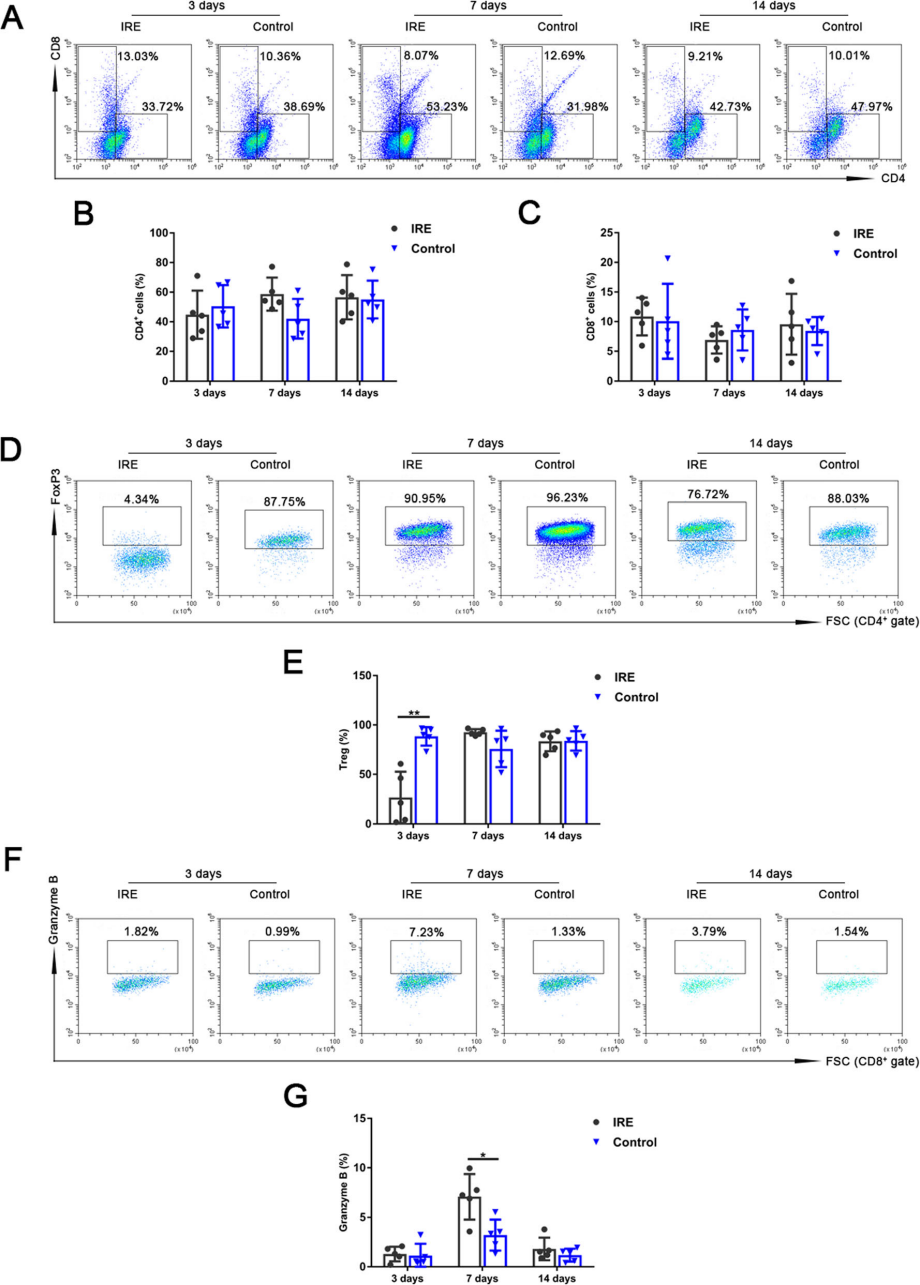
The percentages of tumor-infiltrating T lymphocytes of mice in the IRE and control groups 3, 7, and 14 days post-IRE, analyzed with flow cytometry. **a** Representative flow cytometry plots showing CD4^+^ and CD8^+^ T cells. **b**, **c** The percentages of CD4^+^ and CD8^+^ T cells. **d** Representative flow cytometry plots showing regulatory T cells (Treg) (CD4^+^FoxP3^+^). **e** The percentage of Treg. **f** Representative flow cytometry plots showing cytotoxic CD8^+^ T cells (CD8^+^Granzyme B^+^). **g** The percentage of cytotoxic CD8^+^ T cells. ^*^p < 0.05, ^**^p < 0.01

The results of flow cytometry analysis revealed that CD4^+^ T lymphocytes increased in the IRE group on day 7 and day 14, and CD8^+^ T lymphocytes increased on day 3 and day 14 (Fig. 4a-c). We observed significantly lower Treg cells (CD4^+^FoxP3^+^) (26.9 ± 25.9 vs 88.5 ± 9.4%, *p* < 0.01) on day 3 (Fig. 4d, e) and remarkably higher cytotoxic CD8^+^ T cells (CD8^+^Granzyme B^+^) (7.1 ± 2.3 vs 3.2 ± 1.6%, *p* < 0.05) on day 7 (Fig. 4f, g**)** in the IRE group than those in the control group.

**Fig. 5:**
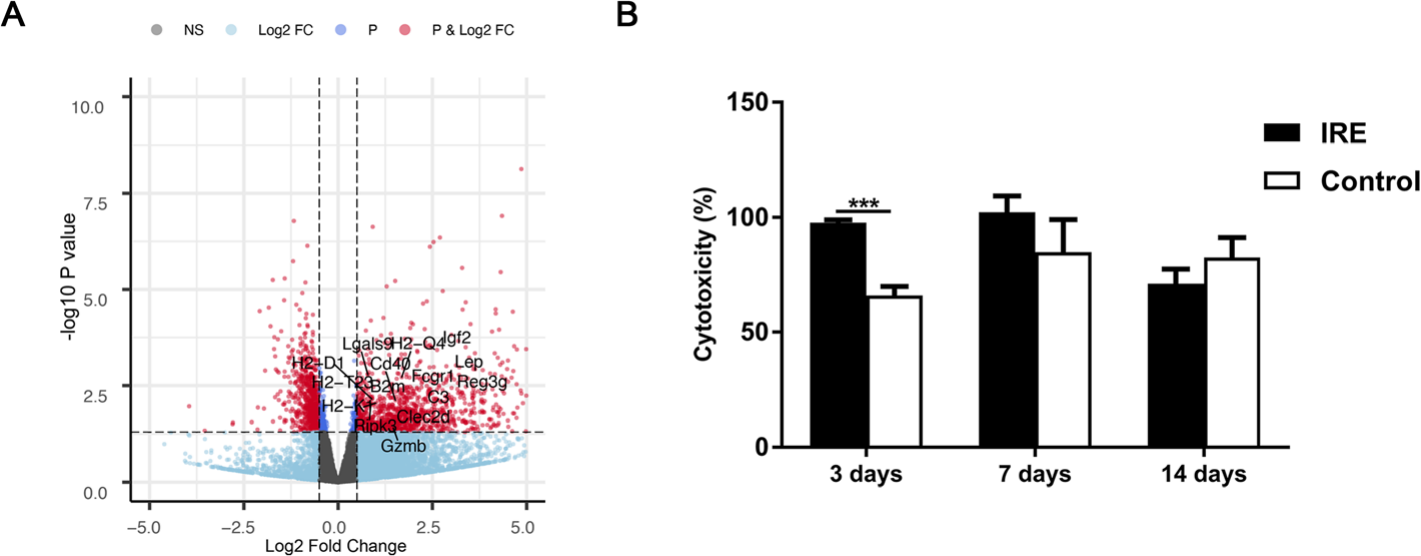
Cytotoxic activity of T cells from tumors of mice in the IRE and control groups. **a** Volcano plots displaying cytotoxicity associated genes, red plots represent DEGs. **b** Cytotoxicity activity of T cells were analyzed by Lactate dehydrogenase (LDH) cytotoxicity assay

Gene expression profiling also revealed a significant increase in T cell mediated cytotoxicity associated genes (Gzmb) in the tumor post-IRE treatment (Fig. 5a), which was consistent with the results of flow cytometry analysis (Fig. 4f, g**)**. To further confirm whether IRE treatment induced specific antitumor immunity, we analyzed cytotoxic activity of T lymphocytes. Antitumor cytotoxicity activity of T cells treated with IRE was higher than that of the control group (97.8 ± 1.2 vs 66.0 ± 3.9%, *p* < 0.001) on day 3 (Fig. 5b). All above results confirmed specific antitumor immunity induced by IRE.

### CD8^+^ T cells infiltrated predominantly at margins but CD4^+^ T cells also infiltrated into the mice tumor after IRE

**Fig. 6:**
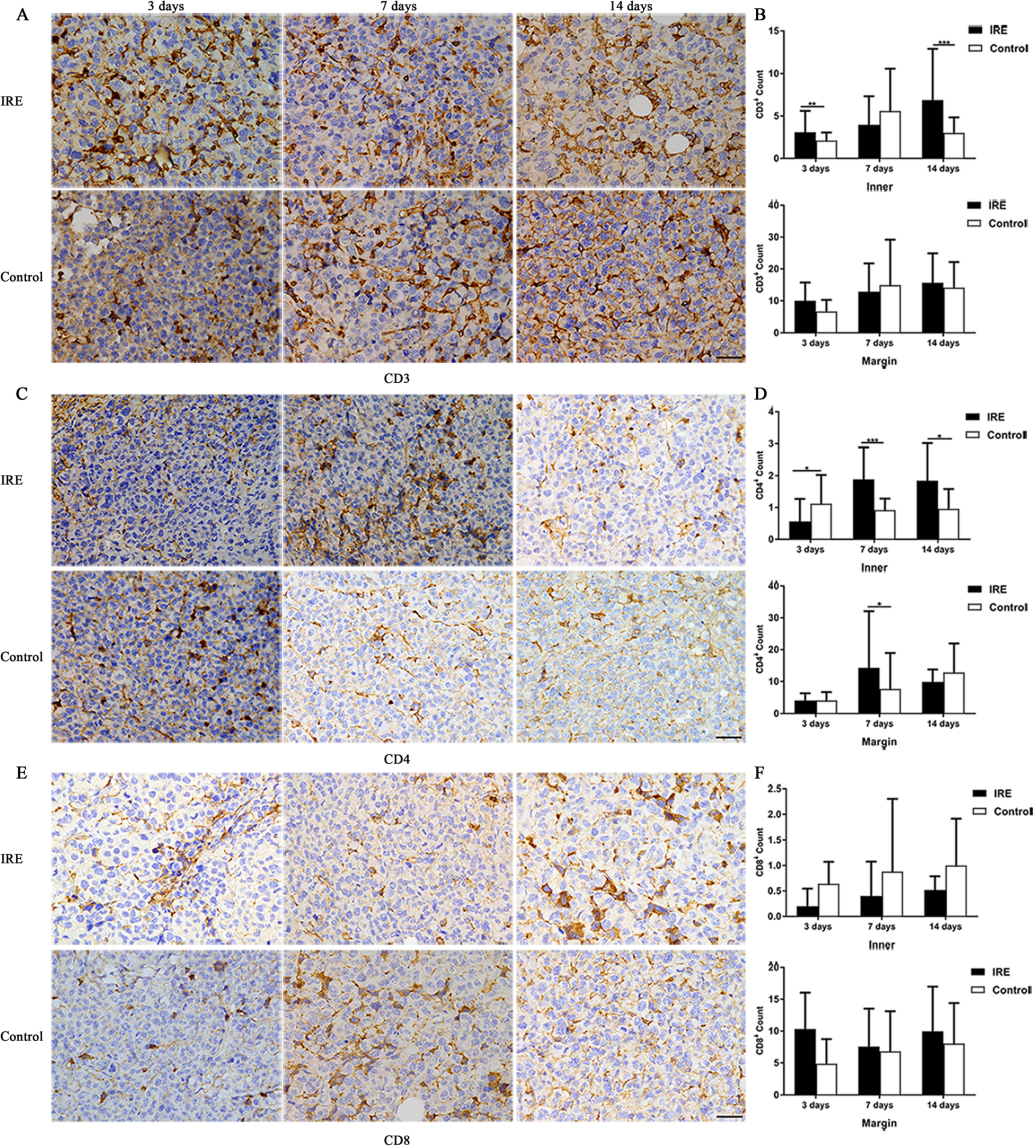
Tumor-infiltrating T lymphocytes counts of mice in the IRE and control groups, 3, 7, and 14 days post-IRE, analyzed with immunohistochemistry. **a**, **c**, **e** Representative micrographs showing cells stained with CD3, CD4, and CD8. Scale bars = 20 μm. **b**, **d**, **f** The numbers of CD3^+^ T cells (**b**), CD4^+^ T cells (**d**), and CD8^+^ T cells (**f**) that infiltrated into the inner regions and at the margin of tumors. ^*^p < 0.05, ^**^p < 0.01, ^***^p < 0.001

The immunohistochemistry results revealed that while the infiltration of CD3^+^ (Fig. 6a, b) and CD4^+^ T lymphocytes (Fig. 6c, d) was intense at the margin, they were also found in the center of tumors. In the IRE group, the number of CD3^+^ T lymphocytes significantly higher than the control group on day 3 (3.1 ± 2.5 vs 2.1 ± 1.0, *p* < 0.01) and day 14 (6.9 ± 6.0 vs 3.0 ± 1.8, *p* < 0.001) in the center of tumors, and CD4^+^ T lymphocytes on day 7 (1.9 ± 1.0 vs 0.9 ± 0.4, *p* < 0.001) and day 14 (1.8 ± 1.2 vs 1.0 ± 0.6, *p* < 0.05). However, we only found infiltration of CD8^+^ T lymphocytes (Fig. 6e, f) at the margins, but scarcely in the center. Although it did not reach a statistically significant level, we did find that in IRE group, there were higher numbers of infiltrating CD8^+^ T lymphocytes in the margin of tumors at different time points compared to the control group (10.3 ± 5.7 vs 4.9 ± 3.9; 7.6 ± 6.0 vs 6.8 ± 6.3; 10.0 ± 7.0 vs 8.0 ± 6.4, respectively).

### IFN-γ increased in the serum of tumor bearing mice after IRE

**Fig. 7:**
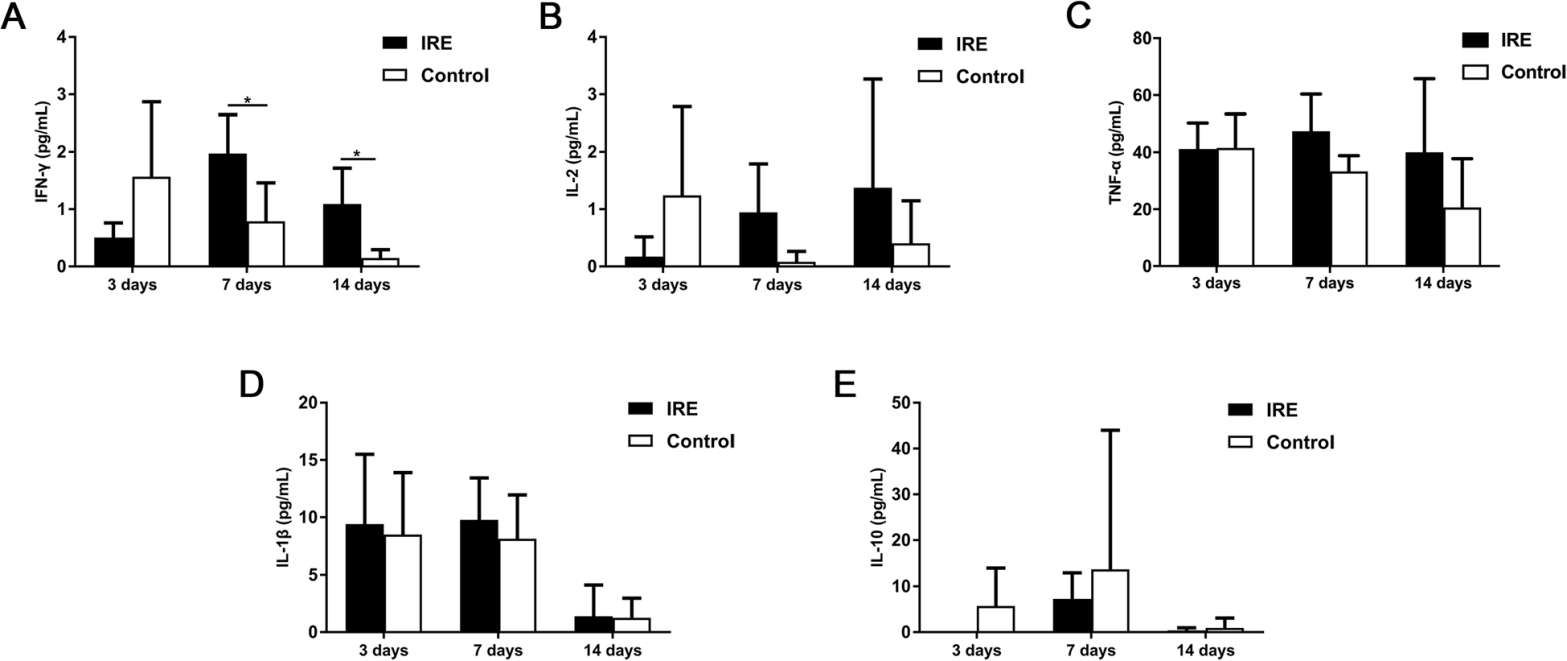
The concentration of serum cytokines in the mice in the IRE and control groups, 3, 7, and 14 days post-IRE: **a** IFN-γ, **b** IL-2, **c** TNF-α, **d** IL-1β, and **e** IL-10. ^*^p < 0.05

Furthermore, we observed a significant increase in the expression profiles associated with cytokine production, predominantly IFN-γ-mediated signaling pathway. Tumor-bearing mice in the IRE group also showed significantly higher serum level of IFN-γ (Fig. 7a) on both day 7 and 14, in comparison with the control group (2.0 ± 0.7 vs 0.8 ± 0.7, *p* < 0.05; 1.1 ± 0.6 vs 0.1 ± 0.1, *p* < 0.05, respectively). Although not statistically significant, we found that the serum levels of IL-2, TNF-α, and IL-1β in the mice treated with IRE were higher than the levels in the control group (Fig. 7b-d). The elevated levels of these would help increase anti-tumor immunity in the body. In addition, the IL-10 level in the IRE group decreased at all time points after IRE treatment (Fig. 7e).

## Discussion

IRE is a novel, non-thermal ablation modality, which was used to treat tumors. Unlike with surgical resection, the ablated tumor tissues are not removed, apoptotic or necrotic cells release damage-associated molecular patterns, such as HMGB1, ATP, ROS and calreticulin, which may serve to achieve in situ tumor vaccination [21]. As a result, antitumor immune response may be induced that in turn can effectuate regression of distant and metastatic lesions, increase the efficacy of IRE. In this study, we investigated the immunological response post-ablation of IRE in patients with HCC, as well as in mice bearing tumors, to provide more evidence for clinical tumor treatment.

For HCC patients, the immune response induced by treatment is complicated. The systemic treatment or collateral events related to treatment can modify the immune response. All patients in this study didn’t receive any systemic treatments before and after IRE treatment, so the immune response observed in this study were thought to be related to IRE treatment.

First, we tested whether IRE induced a systemic immunological response in patients with HCC. Shortly after IRE treatment, an increase in WBC, neutrophil and monocyte counts were observed in the peripheral blood. Neutrophils are the predominant circulating leukocyte population and constitute an important part of the innate immunity. As for monocytes, Sugimoto et al. found a significant early increase in macrophage migration inhibitory factor, followed by rapidly mobilizing of monocytes from the peripheral blood to the ablation zone after IRE, but not RFA, which may facilitate the early reparative process and result in shrinkage of ablation zone [22]. As another key effector of the first-line defense against tumors, NK cells were also found to be elevated post-IRE. NK cells kill tumor cells through the cytotoxic activity without any specific antigen stimulation; they can also regulate innate and adaptive immunity by secreting cytokines and chemokines [23]. The major cytokine produced by NK cells is IFN-γ, which mediates the induction of T helper 1 (Th1) cells, which are associated with a good prognosis of patients with cancer [24]. Indeed, we did found that IFN-γ increased in the serum of mice bearing tumors after IRE. Our results showed that IRE indeed could induce an immediate innate immune response characterized by the increase of neutrophils, monocytes and NK in patients, which may help the patients to reconstruct anti-tumor immunity.

Increasing evidence shows that elevated NLR is a prognostic indicator of mortality [25, 26]. In our patient study, although NLR was found to be elevated significantly 1 day post-IRE, it recovered to the base level within 1 week. This transient elevation may represent a relative lymphocytopenia, capacity of NLR to decline for a short period, reflecting host immunomodulatory activity. Indeed, we observed that IRE caused short-term depletion of circulating CD4^+^ T lymphocytes (activated and memory subsets), but not CD8^+^ T cells. Lymphopenia has been explored in patients with cancer undergoing chemoradiotherapy [27, 28]. Some reports have shown that immune reconstruction following lymphopenia shifts T subsets toward a predominance of activated T cells, enhancing antitumor immunity [29]. Another explanation is the depletion of Treg cells, which are produced in the thymus or induced in the periphery from naïve T cells [30]. Pandit et al. compared the magnitude of decreased Treg cells and the highest longitudinal changes were observed early post-IRE procedure (day 3–5) compared with surgical resection [31]. Chaobin He et al. found a similar changes of Treg cells which increased at day 3 and decreased at intervals of day 3–7 post-IRE [32]. Furthermore, Scheffer et al. detected the levels of Treg cells at delayed time (2 weeks) post-IRE treatment, and a significant decrease was also found [33]. With a longer follow-up time in our study, similar findings showed a transitory increase of Tregs by 3 days followed by a decrease until 2 weeks post-IRE, accompanied by a remarkable increase of activated T cells; after 1 month Treg cells appear to be recovering, which increased significantly. Above results indicated the immunomodulatory effect of IRE with decreased immunosuppressive Treg cells and expansion of effective T cells. However, further investigations are needed to ascertain the long-term antitumor immunity and to evaluate how these immune responses impact the prognosis of patients with various cancer phenotypes.

Apart from systemic antitumor immunity, tumor-infiltrating lymphocytes are associated with favorable prognostic effect [34, 35]. Especially, the introduction of immunoscore has gained significance for the classification of cancers and aid in predicting the outcomes of treatments [36, 37]. Our tumor model study revealed that IRE treatment induced adaptive antitumor immunity within the ablative tumors. At first, we performed RNA sequencing analysis and the results indicated that several adaptive immune process related pathways were up-regulated in the IRE treatment group and several pathways, such as signaling by TGF-beta Receptor Complex were down-regulated. Gene expression profiling also revealed a significant increase in T cell mediated cytotoxicity associated genes (Gzmb) in the tumor post-IRE treatment. Then flow cytometry analysis were performed to further confirm that CD4^+^ and CD8^+^ T cells (CD8^+^Granzyme B^+^) increased, and Tregs decreased in the tumors after IRE. The results of LDH analysis revealed that IRE induced higher antitumor cytotoxicity activity of T cells. These results were consistent with the results of RNA sequencing. All above results together revealed that IRE treatment induced adaptive antitumor immunity within the ablative tumors, dominated by increased cytotoxic CD8^+^ T cells and reduced Treg cells. It is well known that cytotoxic CD8^+^ T lymphocytes are crucial components of tumor-specific cellular adaptive immunity, and they produce perforin, granzyme, or TNF, IFN-γ to kill tumor cells or induce apoptosis [38, 39]. Consistently, serum cytokines, predominantly IFN-γ, were found to be increased post-IRE. However, CD8^+^ T cells infiltrated merely the margin of tumors in both IRE and control groups, which is because immune cells already inside the tumor are probably destroyed by IRE pulses, whereas, CD4^+^ T cells infiltrated both in the center and margin of the same tumor. This difference could be contributed by regional heterogeneity of tumor architecture and tumor antigens. The combined data of our systemic and local antitumor immunity induced by IRE proposed that from 3 days to 2 weeks post-IRE maybe an ideal treatment window for immunotherapy by increasing the effective T cells and decreasing Tregs, resulting in control recurrence and metastasis of ablation therapy. Recent literature reported that IRE combined with immune checkpoint blockade enhanced antitumor immune response, and help overcome the immunosuppressive tumor microenvironment of pancreatic cancer [40, 41]. Therefore, IRE may be an effective modality to overcome the immunosuppressive “cold” tumor microenvironment. However, the immune mechanism induced by IRE needs more in-depth studies. Furthermore, an optimal combination therapy requires validation in animal and patients with HCC, such as IRE combined with immune checkpoint inhibitors, which may improve the prognosis of patients.

## Conclusions

In conclusion, in the current study, we preliminarily investigated the immune activity caused by tumor ablation with IRE in both patients with HCC and tumor-bearing mice. The results demonstrated that IRE upregulated activated T cells and downregulated Tregs in the peripheral blood, which are a benefit for enhancing the sustained anti-tumor activity of patients. Meanwhile, the results from the animal model indicated that IRE could rapidly inhibit local tumor growth by inducing the infiltration of CD4^+^ and CD8^+^ T cells, predominantly cytotoxic CD8^+^ T cells, accompanied by the decrease of Tregs.

## Data Availability

The datasets used and/or analyzed during the current study are available from the corresponding author on reasonable request.

## Abbreviations

CBA: Cytometric bead array
DAB: Diaminobenzidine
DEGs: Differentially expressed genes
ECG: Electrocardiogram
GO: Gene ontology
HCC: Hepatocellular carcinoma
IRE: Irreversible electroporation
LDH: Lactate dehydrogenase
NK: Natural killer
NLR: Neutrophil to lymphocyte ratio
Treg: Regulatory T
Th1: T helper 1
